# The Application Potential of Artificial Intelligence and Numerical Simulation in the Research and Formulation Design of Drilling Fluid Gel Performance

**DOI:** 10.3390/gels10060403

**Published:** 2024-06-17

**Authors:** Keming Sheng, Yinbo He, Mingliang Du, Guancheng Jiang

**Affiliations:** 1 College of Information Science and Engineering/College of Artificial Intelligence, China University of Petroleum (Beijing), Beijing 102249, China; pluto_134340@yeah.net; 2 College of Petroleum Engineering, China University of Petroleum (Beijing), Beijing 102249, China; dml5693@student.cup.edu.cn; 3 National Engineering Research Center of Oil & Gas Drilling and Completion Technology, Beijing 102249, China

**Keywords:** weak gel, neural network, deep learning, molecular mimicry, performance optimization, inflow fluid

## Abstract

Drilling fluid is pivotal for efficient drilling. However, the gelation performance of drilling fluids is influenced by various complex factors, and traditional methods are inefficient and costly. Artificial intelligence and numerical simulation technologies have become transformative tools in various disciplines. This work reviews the application of four artificial intelligence techniques—expert systems, artificial neural networks (ANNs), support vector machines (SVMs), and genetic algorithms—and three numerical simulation techniques—computational fluid dynamics (CFD) simulations, molecular dynamics (MD) simulations, and Monte Carlo simulations—in drilling fluid design and performance optimization. It analyzes the current issues in these studies, pointing out that challenges in applying these two technologies to drilling fluid gelation performance research include difficulties in obtaining field data and overly idealized model assumptions. From the literature review, it can be estimated that 52.0% of the papers are related to ANNs. Leakage issues are the primary concern for practitioners studying drilling fluid gelation performance, accounting for over 17% of research in this area. Based on this, and in conjunction with the technical requirements of drilling fluids and the development needs of drilling intelligence theory, three development directions are proposed: (1) Emphasize feature engineering and data preprocessing to explore the application potential of interpretable artificial intelligence. (2) Establish channels for open access to data or large-scale oil and gas field databases. (3) Conduct in-depth numerical simulation research focusing on the microscopic details of the spatial network structure of drilling fluids, reducing or even eliminating data dependence.

## 1. Introduction

Drilling fluid is often referred to as the “lifeblood” of drilling engineering, serving functions such as cooling and lubricating the drill bit and tools, supporting and stabilizing the wellbore, balancing formation pressure, and transporting and suspending cuttings [1]. Drilling fluids can be classified into three types based on the dispersing medium: water-based, oil-based, and gas-based fluids [2]. Early drilling fluids had simple chemical compositions and lacked functionality. The introduction of polymer science, nanoscience [3], colloid and interface chemistry, and supramolecular chemistry has diversified the types of additives, enhancing the specificity and functionality of drilling fluids [4,5,6,7]. As a dispersed system, the performance of drilling fluids stems from interactions between chemical substances, primarily non-covalent bonds. These bonds can sensitively respond to external environmental stimuli (temperature, pressure, salinity, pH, mechanical forces, and field forces), which vary across different drilling environments. Practice has shown that high-performance drilling fluids can be considered weak gel-like fluids. Their gel properties are crucial for carrying and suspending solid particles, sealing formation fractures, stabilizing the wellbore, reducing downhole losses, minimizing pressure loss, and controlling equivalent circulating density (ECD) [8]. The addition of clay to drilling fluids forms a spatial network structure (gel structure) via non-covalent interactions, ensuring effective cuttings transport and wellbore cleaning during circulation. When drilling fluids enter formation fractures, transitioning from a high-shear flow state to a low-shear or static state, rapid gel formation can effectively seal, reduce pressure transmission, stabilize the wellbore, prevent well collapse, and significantly increase flow resistance, thereby slowing the movement of drilling fluid within fractures and reducing downhole losses.

In recent years, research aimed at improving the gel properties of drilling fluids or using gel materials to address technical challenges in drilling fluids has proliferated. This research can be categorized into two main types: gel-based drilling fluid systems and gel leak prevention and plugging technologies. The first type involves the use of various modified natural materials (such as starch [9], cellulose [10], lignin [11], and xanthan gum [12]) and synthetic polymers (such as reverse emulsion gel microspheres synthesized for oil-based drilling fluid leak prevention and plugging by Zhengqiang et al. [13]) to adjust the gel properties of drilling fluids [14]. The second type involves using gel materials (such as polymer gels [15,16] and superabsorbent resins [17]) to form solid plugs in loss channels for sealing purposes. The relationship between gel performance in drilling fluids, their behavior in the wellbore, and drilling parameters involves complex interactions [18]. Gaining insights from these parameters and developing their mapping relationships is challenging, requiring advanced mathematical modeling techniques combined with human intuition and experience. With drilling targets expanding from shallow to deep layers, from conventional to unconventional oil and gas resources (such as shale oil and gas, tight oil and gas, coalbed methane, and natural gas hydrates), and new energy sources (such as geothermal energy), the drilling industry faces increasingly complex subsurface conditions. These complexities make it difficult to accurately predict formation temperature and pressure systems, rock composition and structure, fluid distribution, and properties, leading to blind decisions in selecting drilling fluid types and additives, designing formulations, and optimizing performance. Consequently, drilling complications such as wellbore instability, stuck pipe, lost circulation, poor hole cleaning, and formation damage frequently occur [19].

Computer technologies, exemplified by artificial intelligence (AI) and numerical computation, are the core drivers of the Fourth Industrial Revolution [20]. Digital and intelligent upgrades of traditional industries are essential to seize new opportunities in industrial transformation and keep pace with the technological revolution. Compared to experimental design, numerical simulation, and AI technologies can provide satisfactory solutions without high experimental costs [21]. Despite the existence of numerous mathematical models for predicting the rheology of non-Newtonian fluids, invasion depth, and hole-cleaning capacity, existing mathematical models have narrow applicability, ideal conditions, and complex structures [22] and cannot effectively predict field data. AI technology has achieved significant research outcomes in recent years, with various AI-based predictive models widely recognized for their ability to handle nonlinear problems [23]. AI technology has been applied in all stages of oil and gas exploration and development, including reservoir modeling and characterization [24,25,26], drilling and production optimization [27,28], and enhanced recovery scheme selection [29]. In the drilling fluid industry, combining numerical simulation and AI technologies with the expertise of drilling fluid specialists can significantly improve the design and optimization of drilling fluids [30,31,32,33]. According to the existing literature, research on the design and performance optimization of drilling fluids has emerged [34]. However, due to the widespread difficulty in obtaining data in the drilling fluid industry, the global understanding of intelligent drilling fluid processes is still in its infancy. In-depth research on intelligent construction methods for drilling fluid systems is urgently needed to promote the development of intelligent drilling fluid theories and technologies.

This paper first introduces the current applications of AI in drilling fluid engineering, emphasizing various research findings proposed by different authors, and outlines the advantages and disadvantages of AI methods in drilling fluid design and performance optimization. Then, the application of numerical simulation technology in drilling fluid engineering is reviewed and summarized, with an in-depth analysis of the current issues and future challenges in the digitization of drilling fluid engineering, aiming to assist subsequent researchers in developing more efficient additives and creating more comprehensive drilling fluid systems.

## 2. Application of Artificial Intelligence Technology in Drilling Fluid Gel Performance Research and Formulation Design

**Figure 1:**
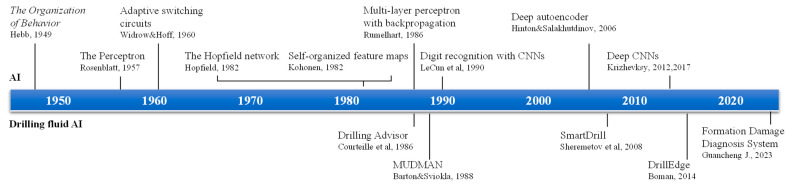
Timeline of artificial intelligence and related applications in drilling fluid [38,39,40,41,42,43,44,45,46,47,49,50,51,52,53].

The term “intelligence” in artificial intelligence (AI) originated from the field of psychology [35]. In 1943, neurophysiologist Warren McCulloch and mathematician Walter Pitts created a neural network model using circuits to explain how neurons function [36]. The term “artificial intelligence” was first coined during a conference at Dartmouth College in the summer of 1956, attended by John McCarthy along with a group of mathematicians, computer scientists, psychologists, neurophysiologists, and other scientists [37]. This marked the official birth of AI as an emerging discipline. Research on AI algorithms intensified in the 1980s (Figure 1). Among these pioneering works [38,39,40,41,42,43,44,45,46,47], the most influential was the 1986 paper by Rumelhart, Hinton, and Williams [43], which described the backpropagation method for training multilayer networks. Thanks to the immense processing power and parallelism of modern graphic processing units (GPUs) and the availability of large visual datasets, deep learning algorithms have achieved numerous breakthroughs, becoming the standard algorithms for visual data processing [48]. This section focuses on the application of AI technology in the design and performance optimization of drilling fluids in the drilling fluid industry.

### 2.1. Expert Systems

Expert systems are AI programs that achieve expert-level problem-solving abilities by representing and processing the knowledge used by human experts. In the early stages of AI development in the oil industry, expert systems were the primary AI technology used, applied to manage drilling operations and address drilling problems [49]. In 1988, Baroid Fluid Services developed MUDMAN to assist field engineers in analyzing 20 parameters of drilling fluids, such as viscosity, density, and clay content, and recommend potential adjustments to address issues with the current drilling fluid. At a North Sea location, MUDMAN correctly diagnosed a drilling fluid contamination problem that had been misdiagnosed by human experts for over a decade [50]. Xiong and Holditch [54] proposed an integrated fuzzy expert system to diagnose reservoir damage and allocate appropriate remediation strategies. Bartko et al. [55] and Nitters et al. [56] combined fuzzy rules with mathematical models to develop structured expert systems for diagnosing and selecting remediation strategies for reservoir damage. In recent years, companies in the oil and gas industry have been actively integrating various AI technologies to build hybrid intelligent systems capable of handling complex processes. Sheremetov et al. [51] reported on “Smart-Drill”, a fuzzy expert system that combines fuzzy logic and web services to diagnose and resolve drilling fluid loss issues, aiding in the development of new plugging gels. Verdende Technologies developed DrillEdge [52], a case-based reasoning software that can identify potential drilling problems and drilling fluid-related issues involving drilling equipment. Cristofaro et al. [57] reported an AI strategy based on supervised learning that collected and generalized data from 500 fluid loss incidents over three years, creating a human–computer interaction interface for field engineers without machine learning knowledge. This AI strategy was validated and applied in two real cases in the Santos Basin, Brazil, saving six days of well construction time. A recent research achievement in the drilling fluid industry was reported in 2023 by Guancheng J. et al. [53]. They proposed a system using conformal transformation, membership analysis, and deep neural networks to study damage removal measures for reservoirs in complex structured wells such as horizontal wells. This system can recommend optimal drilling fluid formulations with damage removal functions based on formation oil parameters, formation fluids, and reservoir physical parameters.

### 2.2. Artificial Neural Networks

Artificial neural networks (ANNs) are AI methods that describe the intelligent behavior of the human brain at a microscopic level, offering strong capabilities in solving fuzzy relations and nonlinear problems [58]. The mathematical principles and parallel computing modeling processes of neural networks are well articulated in P. Zeng’s paper [59], which also explores the convergence and stability of neural networks.

Table 1 summarizes the applications of ANNs in the drilling fluid industry over the past 20 years. These applications cover several areas in the drilling fluid industry, including (a) loss prevention and reservoir protection, (b) prediction of static density and equivalent circulating density, (c) prediction and regulation of rheology, (d) control of fluid loss and prediction of mud cake permeability, (e) wellbore cleaning and cuttings carrying capacity regulation, (f) prediction of hydraulic losses, and (g) formulation design and adjustment of drilling fluids. Table 1 highlights the research content, the ANN methods used, the network architecture, and the evaluation metrics employed in each study. The evaluation metrics include the correlation coefficient R2 (Equation (1)), root mean square error (RMSE, Equation (2)), and average absolute percent relative error (AAPE, Equation (3)). The following points can be summarized from these studies:

1. There are two primary sources of data used to create neural networks: datasets obtained from existing literature and data collected via physical simulations.
2. The most frequently applied areas of ANNs are rheology (including flow patterns) and loss prediction, accounting for 39% and 18%, respectively. Loss prediction is critical for controlling the cost of drilling fluids, while rheology is a crucial performance characteristic of drilling fluids, directly reflecting their ability to carry and suspend cuttings and solid particles like barite.
3. Most researchers using ANNs have a single neuron in the output layer. However, the works of Jeirani [60], Elkatatny [61], Xianzhi [62], and Arash [63] involved 2, 4, 2, and 7 outputs, respectively, indicating that ANNs can handle multi-output parameter prediction tasks.
4. The most popular evaluation metric is R^2^, although some studies have used mean squared error (MSE), and one study [64] used the correlation coefficient R. For consistency and readability, these were converted to RMSE and R^2^. Overall, these models demonstrate excellent performance based on the evaluation metrics.

(1) $$R^{2}=1-\frac{\sum_{i}{\left({\hat{y}}_{i}-y_{i}\right)}^{2}}{\sum_{i}{\left({\bar{y}}_{i}-y_{i}\right)}^{2}}$$

(2) $$\mathrm{RMSE}=\sqrt{\frac{1}{m}\sum_{i=1}^{m}{\left({\hat{y}}_{i}-y_{i}\right)}^{2}}$$

(3) $$\mathrm{AAPE}=\frac{1}{m}\sum_{i=1}^{m}\frac{\left|{\hat{y}}_{i}-y_{i}\right|}{y_{i}}\times 100\%$$

**Table 1:** Summary of research on drilling fluid industry to which ANN was applied.

Author (s)	Type of Study Conducted	ANN Method and Architecture	Performance Evaluation Criteria		
			RMSE	AAPE	R^2^
Ozbayoglu, Mehmet (2002) [65]	The thickness of shale beds is computed to adjust the rock-carrying capacity of drilling fluids	-	-	25%	-
Osman, Aggour (2003) [66]	Drilling fluid density is predicted	BP 4-6-1	0.0056	3.67%	0.9998
Jeirani, Mohebbi (2006) [60]	Drilling fluid loss and mud cake permeability are forecasted to adjust filtration wall-building properties	BP 4-30-2	-	-	Filter volume: 0.9815, cake permeability: 0.9433
E. M. Ozbayoglu, M. A. Ozbayoglu (2009) [67]	Drilling fluid flow patterns are fitted, and drilling fluid hydraulic losses are calculated	BP, Jordan/Elman	FP with BP: 0.0774, FP with J/E: 0.0707, FPL with BP: 0.0707, FPL with J/E: 0.0707	-	-
Yongbin, Yeli et al. (2009) [68]	Computer software for designing drilling fluids based on specified rheological properties is developed	BP	-	-	-
Moazzeni et al. (2011) [69]	Circulation losses are forecasted	FFBPN	-	-	0.7654
Olutayo, Theodore [70]	Annular fluid flow patterns are predicted	FFBPN 6-26-1	-	-	0.9028
Rooki et al. (2012) [71]	The settling velocities of solid particles in both Newtonian and non-Newtonian fluids are calculated	FFBPN 6-12-1	0.0380	-	0.9860
Moazzeni et al. (2012) [72]	Circulation losses are predicted	FFBPN 18-30-1	-	-	0.82
Razi et al. (2013) [73]	The apparent viscosity of drilling fluids is forecasted	FFMLP 3-3-1	-	-	0.993
Razi et al. (2014) [74]	The rheological properties of xanthan gum are predicted	FFBPN 3-5-1	0.4960	-	0.9980
Rooki et al. (2014) [75]	The hole-cleaning capability of foam drilling fluids is forecasted	FFBPN 6-10-1	-	5.93%	0.914
Reza Jahanbakhshi et al. (2014) [76]	Circulation losses are predicted	MLP 11-3-1	51.9427	-	0.94
Rooki (2015) [77]	Fluid pressure losses are forecasted	FFMLP 6-10-1	-	4.32%	0.999
Chen et al. (2015) [78]	The equivalent static density of drilling fluids is predicted	BP	-	2.00%	-
Elkatatny et al. (2016) [61]	Predicting the rheological properties of invert emulsions to assist in designing shear-thinning weak gels	4-12-4	-	-	n: 0.954, PV: 0.917, K: 0.9205
Xianzhi et al. (2016) [62]	Washout fluid flow rates are optimized	BP 7-10-2	0.0768	-	1
Barati-Harooni et al. (2016) [79]	Frictional pressure losses are forecasted	RBF	0.008783	-	0.9965
Reza (2016) [80]	Hydraulic losses of drilling fluids are predicted	GRNN 6-260-1	8.45 kPa	6.24%	0.98
Khim et al. (2016) [81]	Drilling fluid flow rates are forecasted	RNN	-	5.6%	-
Behnoud far, Hosseini (2017) [82]	Circulation losses are predicted	FFBPN 4-3-1	0.1879	-	0.9991
Vitor Diego et al. (2017) [83]	The impact of xanthan gum, bentonite, and barite dosage on the apparent viscosity of drilling fluids is evaluated	FFBPN 4-6-1	2.7803cP^2^	-	-
Arash, Mohammadali (2017) [63]	Drilling fluid design is conducted	BP 3-6-7	-	-	-
Elkatatny (2018) [84]	The relationship between drilling fluids and reservoir permeability is established to optimize reservoir protection performance	BP 8-20-1	-	5.6%	0.99
Al-Khdheeawi, Mahdi (2019) [85]	The apparent viscosity of drilling fluids is predicted	BP	-	10.9%	0.988
Golsefatan, Shahbzi (2020) [86]	The effect of nanoparticle concentration on drilling fluid loss is forecasted	BP	0.6926	2.6636	0.9928
Ahmed et al. (2021) [87]	Drilling fluid rheology is predicted	BP [AV PV]2-42-1, [YP n R300 R600]2-34-1	-	4.81–7.97	0.9–0.95
Di et al. (2022) [88]	The sand content of drilling fluids is calculated	BP 3-10-1	-	0.08%	0.9997
Alkouh (2024) [64]	The plastic viscosity of drilling fluids is forecasted	2-3-1	0.975	-	0.64

### 2.3. Support Vector Machines

**Figure 2:**
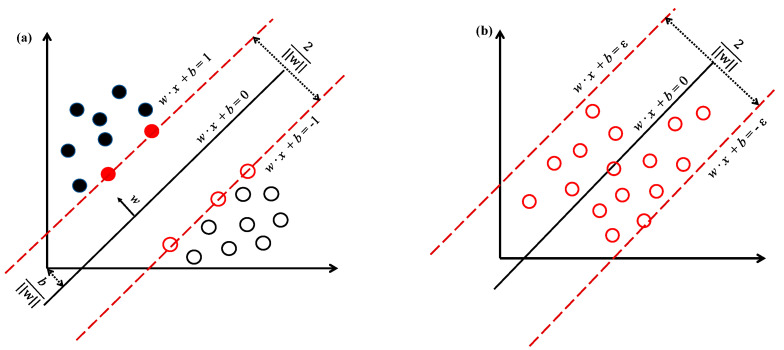
Support vector machine schematic diagram: (**a**) classification; (**b**) regression.

Support vector machine (SVM) was originally a binary classification model, with its basic model being the maximal-margin linear classifier defined in the feature space, as illustrated in Figure 2. For samples that are linearly inseparable in finite-dimensional vector space, mapping them to a higher-dimensional vector space results in the maximal-margin SVM, which is the non-linear SVM. In comparison to neural networks, SVM bears some formal resemblance but substantially differs. Neural networks belong to the category of “black-box” models, optimizing objectives based on empirical risk minimization, yielding unstable training outcomes, often necessitating ample samples. In contrast, SVM boasts a solid mathematical foundation; whether linear or non-linear, its convergence has been rigorously proven mathematically. Following the principle of structural risk minimization, SVM exhibits stronger generalization capabilities compared to neural networks, devoid of involvement in probability measures and the law of large numbers, thus differing from existing statistical methods. Further details on support vector machines can be gleaned from the book by Andreas and Ingo [89].

Table 2 summarizes the applications of support vector machines in the regulation and optimization design of drilling fluid properties. Researchers commonly employ SVM to assist in adjusting drilling fluid rheology, high-temperature, high-pressure density, carrying capacity, predicting circulation loss, and pressure loss. The radial basis function is the most frequently utilized kernel function. The final decision function of SVM is determined by only a few support vectors, and the computational complexity depends on the number of support vectors rather than the dimensionality of the sample space, thus circumventing the curse of dimensionality. Fundamentally, SVM circumvents the traditional process from induction to deduction and instead conducts transductive reasoning from training samples to forecasted samples, presenting an efficient theoretical framework for small-sample statistics, which aligns well with the issues of high-dimensional non-linearities and difficulty in acquiring subsurface data pertinent to drilling fluid formulation design.

### 2.4. Genetic Algorithms

In recent years, metaheuristic algorithms have been applied to solve various engineering problems, with most drawing inspiration from biological evolution processes, swarm behaviors, and physical laws [99]. These nature-inspired algorithms can be broadly classified into two categories: single-solution-based and multi-solution-based metaheuristic algorithms. Single-solution-based metaheuristic algorithms employ a single candidate solution, refining it via local searches, often prone to local optima. Notable single-solution-based metaheuristic algorithms include the Cuckoo Search Algorithm (CSA), simulated annealing, tabu search, microcanonical annealing, and guided local search. On the other hand, multi-solution-based metaheuristic algorithms utilize multiple candidate solutions in each search, maintaining solution diversity to evade local optima. Famous multi-solution-based metaheuristic algorithms comprise Genetic Algorithm (GA), Particle Swarm Optimization (PSO), Imperialist Competitive Algorithm (ICA), Ant Colony Optimization, Spotted Hyena Optimization, Emperor Penguin Optimization, and Seagull Optimization [100], with Genetic Algorithm being the most widely employed in engineering applications.

Genetic Algorithm is an optimization algorithm developed based on the Darwinian assumption. According to this assumption, each possible solution in every iteration is generated by different genetic operators, including mutation and crossover of the preceding solution. After generation, each solution is evaluated by a predefined function, referred to as the fitness function, and the potential solutions are ranked based on the evaluation results. The advantages of the Genetic Algorithm lie in its derivative-free optimization process, widespread applicability, and avoidance of local optima [101]. In 2001, D. Erbas et al. [101] simulated reservoir damage caused by precipitation, adsorption, dissolution, and clay swelling using Genetic Algorithm and material balance theory to adjust the reservoir protection capability of drilling fluids. While this method is straightforward and practical, the factors considered are overly simplistic, failing to systematically assess reservoir damage during oil and gas field development. In 2012, Rooki et al. [102] employed a Genetic Algorithm to determine the rheological parameters of Herschel–Bulkley drilling fluids. In 2014, Barati et al. [103] utilized Multi-Gene Genetic Programming (a branch of Genetic Algorithm) to develop empirical relationships predicting the drag coefficient around smooth spheres, with Goldstein and Giovanni using Genetic Algorithm to predict the settling terminal velocity of solid particles in drilling fluids to adjust their carrying capacity [104]. However, their compiled dataset predominantly focuses on single particle studies, overlooking the settlement of dense sediment suspensions, floc particles, and the influence of particle shape on settling terminal velocity. In 2018, Hocine et al. [105] optimized the rheological parameters of environmentally friendly drilling fluids formulated with Algerian bentonite and two polymers (hydroxyethyl cellulose and polyethylene glycol) using Genetic Algorithm. According to the model results, researchers can adjust the optimal concentration of NaCl to avoid deterioration of rheological parameters due to gelation mechanisms at high temperatures. In 2023, Dipankar and Sigve [106] employed a hybrid approach of Genetic Algorithm and fuzzy logic to estimate the concentration of cuttings in drilling fluids, with Genetic Algorithm optimizing the weights of fuzzy rules. It is noteworthy that this is also a significant application of the Genetic Algorithm. Operating directly on structural objects, Genetic Algorithm does not necessitate differentiation or continuity constraints on functions, possessing inherent implicit parallelism and superior global optimization capability, employing a probabilistic optimization approach to automatically acquire and guide the optimization search space. Therefore, utilizing Genetic Algorithm for large-scale optimization is one of the current frontiers of research.

### 2.5. Hybrid Intelligent Algorithms

As energy extraction ventures delve deeper and geological conditions become increasingly complex, the formulation design of drilling fluids has evolved into a large-scale optimization problem. Faced with such a multimodal, high-dimensional, and constrained multi-objective optimization problem, employing a single intelligent algorithm would be restricted by the inherent limitations of that algorithm. By combining two or more intelligent algorithms (typically with each having unique characteristics) and using them in accordance with certain rules to form hybrid optimization algorithms, one can leverage their strengths while mitigating their weaknesses, achieving more satisfactory results. For instance, neural networks converge slowly and are prone to local optima, whereas the metaheuristic algorithms mentioned in Section 2.4, when combined, such as PSO-ANN and GA-ANN, outperform their individual usage. Table 3 summarizes the application of the hybrid intelligent algorithm approach in solving drilling fluid engineering problems.

## 3. Advantages and Disadvantages of Different Artificial Intelligence Technologies

The paper provides an overview of five artificial intelligence (AI) technologies, each with its unique advantages and inevitable limitations concerning robustness to noise, convergence speed, data scale requirements, generalization capability, and susceptibility to overfitting. Wolpert and Macready proposed the “No free lunch theorem for optimization” [119], suggesting that if all problems have equal difficulty and performance evaluation standards are absolutely fair, all methods used to solve problems would perform equally. No AI method can effectively address all challenges across all data and computational conditions. To demonstrate the effectiveness and stability of applying these AI technologies to the study of drilling fluid gel performance, researchers have compared the application scenarios of two or more AI technologies. Table 4 presents these studies. Comparative analysis reveals that, in the study of drilling fluid gel performance, SVM performs the best, followed by ANN, while FIS is relatively inferior. ANN, SVM, and GA exhibit robustness to noise, with ANN possessing self-organizing capabilities, requiring a large amount of data to predict complex phenomena effectively, whereas support vector machine requires fewer data; combining Genetic Algorithm with other algorithms can enhance global optimization capability.

## 4. Application of Numerical Simulation Technology in Drilling Fluid Gel Performance Research and Formulation Design

Numerical simulation technology is a valuable tool that seamlessly integrates computational science with engineering problems, fundamentally altering the way researchers develop and analyze drilling fluid materials. The basic principle of numerical simulation technology involves deriving corresponding equations based on mathematical and physical laws. Although not all equations globally converge, specific initial and boundary conditions can be set according to engineering problems. By utilizing numerical calculation methods to discretize and solve continuous mathematical–physical equations, discrete field variable distributions approximate analytical solutions to the original equations. The primary numerical computation methods include finite difference method, finite element method, and finite volume method. The finite difference method currently employs Taylor series expansion to construct differences. By combining different differential formats in time and space, different accuracy differential calculation results can be obtained [123]. The finite element method utilizes extremum principles and partition interpolation, selecting approximation functions in variational calculations and integrating regions, exhibiting broad adaptability and standardized discrete equation forms, facilitating programming. However, it fails to provide reasonable physical explanations for requirements such as conservation of percolation, strong convection, and incompressibility in dealing with seepage flow problems, resulting in significant errors [124,125]. On the other hand, the finite volume method assigns a non-overlapping control volume around each grid node of the computational domain, integrating control equations for each control volume to derive discrete equations [126]. The advantages and disadvantages of the three methods are summarized in Table 5.

As mentioned in the introduction of this paper, the complexity of drilling fluids is influenced by factors such as the compatibility of additives, chemical composition, and environmental conditions. This poses challenges to accurately characterize and predict their behavior solely via traditional experimental methods. Taking rheology as an example, parameters characterizing rheology include apparent viscosity, plastic viscosity, dynamic shear force, dynamic plasticity ratio, initial shear force, final shear force, 6 rpm reading, and 3 rpm reading, among others. It is unlikely that we can completely elucidate the impact of changes in any one factor on the overall drilling fluid properties via experimental means, as these factors are not mutually independent. Researchers can utilize numerical simulation technology to explore the complex interactions between key variables such as molecular structure, mechanical force, and fluid dynamics, thereby gaining deeper insights into drilling fluid behavior and formulating wiser drilling fluid formulations. Thanks to the rapid development of modern computational science, the means of numerical simulation are evolving rapidly, such as lattice Boltzmann simulation, molecular simulation, continuous-time stochastic simulation, full-scale pore simulation, etc., gradually transitioning from continuum mechanics (hydrodynamic equations) to more complex probabilistic process numerical systems [127,128]. Numerical simulation technologies applied in the drilling fluid industry include computational fluid dynamics (CFD), molecular dynamics simulation (MD), and Monte Carlo simulation, among others.

### 4.1. Computational Fluid Dynamics

CFD is widely employed to simulate the flow and fluid mechanics behavior of drilling fluids [129,130], aiding researchers in gaining insights into mass transfer phenomena to optimize fluid flow patterns for guiding drilling fluid design. Table 6 reviews the applications of numerical simulation technology in the optimization design of drilling fluids. The researchers’ research focuses, algorithms or software platforms used, discretization methods, and research findings are summarized in Table 6. From this, the following points can be summarized:

1. Researchers utilize CFD to study drilling fluid rheology, circulation loss, carrying capacity, plugging ability, and invasion processes. The most frequently occurring theme is the study of drilling fluid carrying capacity, which is attributed not only to the inherent advantages of the CFD method but also to the difficulty in observing the settling velocity of solid particles under high-temperature and high-pressure conditions.
2. Eulerian method, discrete element method, immersed boundary method, and distributed Lagrange multiplier method.
3. The governing equations used to describe fluid motion are mainly the Navier–Stokes equations or Eulerian–Eulerian equations.

### 4.2. Molecular Dynamics Simulation

Molecular dynamics (MD) can be used to investigate the behavior of individual molecules in the spatial network structure of drilling fluids [153], providing insights into the interaction processes and results of polymer chains, solvent molecules, and solute molecules at the atomic scale. MD can predict the expansion behavior [154] and diffusion rate [155] of drilling fluids. Below are the applications of MD in the drilling fluid industry.

#### 4.2.1. Drilling Fluid Additives

Anderson et al. (2010) [156] simulated the inhibitory effects of various additives in drilling fluids using molecular dynamics methods. They separately studied the interaction between three inhibitor molecules, namely, polyepoxypropane-diamine, polyethylene glycol, and polyepoxyethylene-dipropylene acid ester, with montmorillonite, providing molecular and atomic-level explanations for the interaction patterns between expansion inhibitors and clay minerals. Zihua et al. (2022) [157] conducted a full-atom study on the mechanism of action of modified polyvinyl alcohol as a water-containing sediment wellbore stabilizer on rock matrix surfaces, explaining the adsorption of this wellbore stabilizer on the rock matrix surface and demonstrating that the entanglement of stabilizer molecule chains enhances the interaction force between upper and lower surfaces. Liao et al. (2023) [158] revealed the inhibition mechanism of the natural gas hydrate inhibitor SYZ-2 they developed using MD: SYZ-2 inhibits hydrate nucleation by promoting the aggregation of methane molecules, reducing methane solubility. Jiafang et al. (2023) [159] applied MD to study the adsorption mechanism of cationic polyacrylamide (CPAM), a shale inhibitor in drilling fluids, on the surface of sodium-based montmorillonite, revealing two main adsorption configurations of CPAM: a. adsorption of tertiary amine groups; b. co-adsorption of tertiary amine groups and amide groups. Zonglun et al. (2023) [160] developed an organic borate ester lubricant and used MD to demonstrate that this lubricant can adsorb on the drill string and wellbore surfaces via hydrogen bonding and van der Waals forces.

#### 4.2.2. Drilling Fluid Chemical

**Figure 3:**
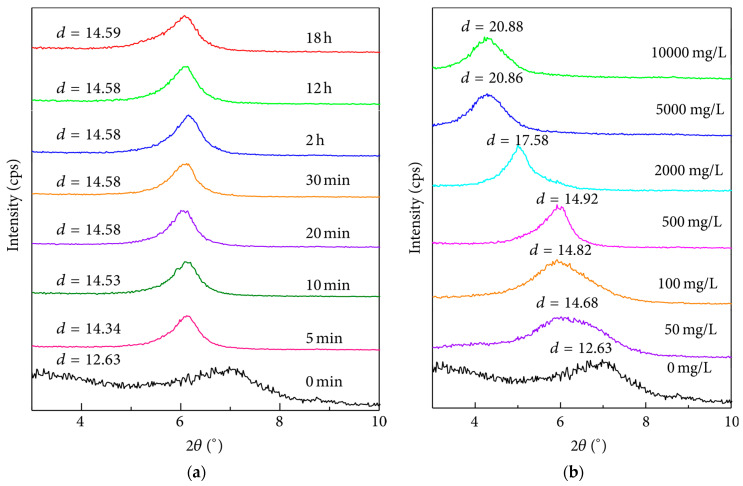
X-ray diffraction patterns of Mt before and after equilibrated at different contact times (**a**) and initial concentrations (mg/L) (**b**) of C12mimCl (/Å) [162].

Wang et al. (2015) [161] utilized MD to study the interlayer microstructure of montmorillonite melted with 1-hexadecyl-3methylimidazolium chloride monohydrate (C_12_minCl). With the exchange of sodium ions and C_12_min^+^, the interlayer space of montmorillonite expanded, leading to a significant increase in the basal spacing d001 of montmorillonite. The XRD analysis results showed that intercalation equilibrium could be reached in 5 min (Figure 3a, the experimental concentration of C_12_min^+^ was 1000 mg/L), and the saturation concentration of C_12_min^+^ was 5000 mg/L (Figure 3b). The research results indicate that the insertion of C_12_minCl into the interlayer space of montmorillonite affects the rheological properties of the drilling fluid system, improving various drilling fluid properties. Jin et al. (2017) [162] used MD to study the mechanism of clay damage, proposing that clay damage occurs via two methods: expansion and instability of the clay structure. The simulation results were consistent with experimental data for sodium-based montmorillonite but not for potassium-based montmorillonite. Xiaohua et al. (2023) [163] investigated the evolution of interfacial friction in oil-based drilling fluid environments using ReaxFF simulation, analyzing molecular motion and friction chemical reactions of oil-based drilling fluid molecules, indicating that the decrease in friction is attributed to fluid dynamic lubrication of oil-based drilling fluid molecules and the passivation of a large amount of H_2_ and a small amount of H atoms produced by friction chemical reactions. Jinbei et al. (2024) [164] used all-atom MD to study the morphology and stability of montmorillonite colloids in a calcium ion environment, providing references for the failure of drilling fluid systems caused by calcium contamination.

#### 4.2.3. Rheological Control of Drilling Fluids

Liao et al. (2022) [165] established a model for natural gas hydrates in clay mineral pores and investigated the impact of drilling fluids on hydrate stability at different intrusion velocities using Molecular Dynamics (MD). This achievement contributes to a better understanding of hydrate decomposition in flow systems and the development of high-performance hydrate drilling fluid materials. Jingping et al. (2022) [166], via MD simulations, infrared spectroscopy, and rheological experiments, elucidated the high-temperature degradation mechanism of drilling fluids: elevated temperatures disrupt the layered structure of clay in drilling fluids, resulting in reduced aluminum elements and hydroxyl groups on the surface, consequently lowering the clay’s negative charge and hydrophilicity. Yunjie et al. (2023) [167] utilized MD simulations to study the shear viscosity variation in supramolecular polymer drilling fluid systems with shear rate. The results indicated that when the shear rate exceeds a critical value, the decrease in shear viscosity is attributed to polymer molecules aligning along the flow field. However, at extremely high shear rates, the reduction in shear viscosity is due to the opening of molecular entanglement structures, leading to the destruction of the fluid’s network structure.

### 4.3. Monte Carlo Methods

Monte Carlo methods are frequently employed to simulate the statistical behavior of drilling fluid systems [168], aiding in predicting the macroscopic properties of drilling fluids based on the behavior of individual molecules or particles. This facilitates the optimization of drilling fluid formulations. Xiaoming et al. (2019) [169] utilized Monte Carlo simulations to investigate the contamination level of coal reservoirs by drilling mud in various-sized fractures. The results indicated significant contamination of fractures of different scales when the initial permeability was low. Grabriel et al. (2021) [170] utilized the Monte Carlo model to assess the uncertainty in the particle size distribution of heavy spar and selected the optimal characteristic particle size.

Some simulations combine multiple techniques. For instance, Albattat et al. (2022) [171] combined Finite Element Method (FEM) and Monte Carlo simulations to evaluate the sensitivity and uncertainty of drilling fluid and underground parameters. Ahmed et al. (2020) [172] conducted geometric optimization of three inhibitors studied using Density Functional Theory (DFT) hybrid functions. They assessed their absolute electronegativity and hardness and corroborated the synthetic surfactant’s adsorption capability on iron crystals via Monte Carlo simulations. The results from both methods mutually validated and aligned with experimental results. These numerical simulation methods expedite the design process, enabling researchers to explore vast design spaces, optimize material performance, and predict drilling fluid performance in various environments. Coupled with experimental techniques, inverse modeling techniques have enhanced simulations and improved prediction accuracy. The fusion of numerical simulation and optimization algorithms also enables researchers to determine the optimal drilling fluid formulations and additives that meet specific performance standards. For example, Dina et al. (2021) [173] employed Monte Carlo simulations to elucidate the impact of non-ionic surfactants on the rheological properties of synthetic-based drilling fluids and used random forest algorithms to reveal the influencing factors of non-ionic surfactant adsorption on organic clays.

## 5. Learnings from the Review

Continuing with the synthesis of artificial intelligence (AI) and numerical simulation applications in drilling fluid gel performance research and formulation design, several observations emerge:

1. A review of AI technologies in drilling fluid processes reveals that approximately 52.0% of studies are related to artificial neural networks (ANNs), 22.6% to support vector machines (SVM), 8.6% to Expert Systems, and 16.7% to Genetic Algorithms.
2. Circulation loss and rheological control in drilling fluids emerge as the most frequently researched topics, indicating significant concern among petroleum industry professionals regarding these issues.
3. The primary obstacle to applying AI in drilling fluid design and optimization is data acquisition. Insufficient data and excessive input parameters often lead to model overfitting.
4. Extensive experimental research has been conducted on the mechanics of drilling fluid carrying cuttings, including studies involving particles of various shapes. However, spherical particles remain the predominant reference in studies of settling velocity.
5. AI technologies offer unique advantages in knowledge acquisition, adaptation to new problems, and handling outliers. Selecting different methods for different drilling fluid optimization issues is necessary.
6. For high-dimensional datasets, careful selection of the most important parameters and removal of redundant information are crucial to enhancing model prediction accuracy. Therefore, feature engineering is particularly important.
7. While different methods have their limitations, combining various AI techniques and even integrating AI with numerical simulation techniques can yield better results.

## 6. Future Perspective

**Figure 4:**
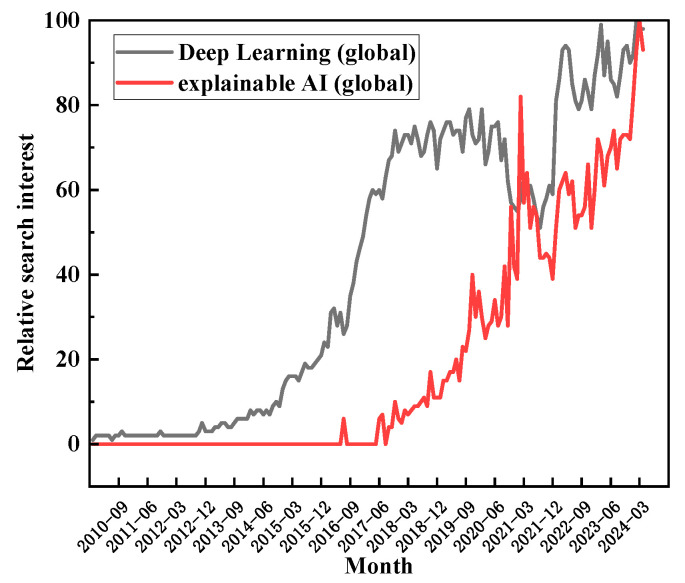
Illustrates the interest evolution towards two terms according to Google Trends.

**Figure 5:**
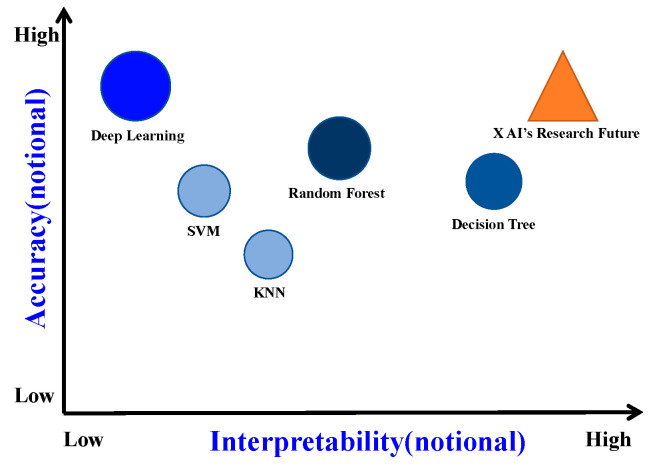
Accuracy versus interpretability for different ML models [174].

1. The rapid growth in the application of advanced AI technologies across various facets of the petroleum industry promises significant economic benefits. Therefore, low transparency and interpretability are clearly unacceptable. A search on Google Trends reveals significant growth in publications related to both “Deep learning” and “Explainable AI” (XAI) over the past 14 years. However, while the curve for deep learning has plateaued over the past 6 years, XAI has experienced exponential growth during the same period (see Figure 4). Enhancing the interpretability of AI will greatly improve the credibility of intelligent models and promote the intelligentization of the drilling fluid industry. Thus, the application of high-precision and interpretable XAI technologies in drilling fluid design and performance optimization is an important research direction (see Figure 5).

2. Geological and formation conditions in oil and gas well completions are complex and variable, making it difficult to accurately and completely obtain downhole data. Purely data-driven AI technologies may not be able to address issues such as quantitative diagnosis of reservoir damage. Moreover, existing numerical simulation techniques often rely on overly simplified assumptions, making it challenging to incorporate the microscopic details of drilling fluid systems. Therefore, there is a research focus on more comprehensive numerical models and small-sample AI technologies for drilling fluid design and performance optimization.

3. While AI and numerical simulation technologies have matured at the technical level, harnessing their full potential requires historical data as a foundation. To benefit the industry, practitioners should open avenues for accessing historical data, thereby enabling the creation of larger and more comprehensive databases, which can help address many challenges.

4. Since solid particles constitute a significant proportion of drilling fluid systems and have vastly different mechanical properties from the liquid phase, solid particles are one of the key factors influencing drilling fluid gel performance. Factors such as solid-phase content, particle size distribution, and particle shape impact the gel performance of drilling fluids, which is an area requiring urgent research. Detailed comparisons facilitated by AI technologies can be employed to analyze the primary controlling factors.

5. Flow of drilling fluids in the wellbore is typically turbulent. Therefore, the consideration of solid-phase concentration and expansion in turbulent conditions, the development of fluid-solid coupling models for drilling fluid gel performance, and the simulation of the impact of turbulence on the transport behavior of a cluster of cuttings rather than individual particles remain areas for future research.

## 7. Conclusions

This paper has reviewed the applications of artificial intelligence (AI) and numerical simulation technologies in the study of drilling fluid gel performance and formulation design. Based on the findings above, the following conclusions can be drawn:

1. Research and formulation design of drilling fluid gel performance present high-dimensional nonlinear problems. When employing numerical simulation and AI technologies, careful consideration should be given to method selection and feature engineering.

2. Neural networks are the most widely utilized AI technology in drilling fluid design and performance optimization. However, this does not imply that other AI technologies cannot be effectively applied in the drilling fluid industry.

3. Via the discussion in this paper, it is evident that no single AI or numerical simulation method can address all issues related to drilling fluid design and performance optimization. Combining one or more of these methods may yield better results than using them individually.

4. Difficulty in acquiring downhole data and poor interpretability are the primary obstacles to the application of AI technologies in drilling fluid design and performance optimization. This challenge can be addressed via the development of Explainable AI (XAI) technologies.

5. The overly idealized assumptions in current numerical simulation technologies applied in the drilling fluid industry represent a limitation.

## Figures and Tables

**Table 2:** Summary of research on drilling fluid industry to which SVM was applied.

Author (s)	Type of Study Conducted	SVM Kernel Function	Performance Evaluation Criteria		
			RMSE	AAPE	R^2^
Olutayo and Theodore (2011) [70]	Predicting the flow patterns of unidirectional flow	using polynomial and Gaussian radial basis functions	-	-	0.9528
Wang et al. (2012) [90]	Forecasting the density of drilling fluids under high-temperature and high-pressure conditions	using radial basis functions	0.117	0.872%	0.9994
Mingzhong et al. (2014) [91]	investigating the influence of wall factors on the rock-carrying capacity of drilling fluids	using radial basis functions	0.0272	-	0.9844
Mehmet et al. (2014) [92]	Predicting the pressure losses of Newtonian and non-Newtonian fluids	-	-	Newton Fluid: 5.1%, Non-Newton Fluid: 5.98%	-
Jahanbakhshi, Keshavarzi (2015) [93]	Forecasting circulation losses	utilizing polynomial kernel functions and Gaussian kernel functions	-	-	0.9851
Shahdi, Arabloo (2016) [94]	Predicting frictional pressure losses	-	-	-	0.99
M. Hoang (2016) [95]	Predicting the viscosity of drilling fluids	using radial basis functions	-	2.7%	-
Chhanty (2017) [96]	Forecasting circulation losses of drilling fluids	utilizing radial basis functions	-	1.61%	0.99
Golsefatan and Shahbazi (2021) [97]	Evaluating the impact of nano-silica on drilling fluid loss	using radial basis functions	0.4588	4.6878%	0.9940
Khim Chhantyal et al. (2021) [98]	Predicting the density of drilling fluids under high-temperature and high-pressure conditions	-	-	-	0.9966
Li Chen (2022) [34]	Optimizing the chemical formulation of drilling fluids	utilizing radial basis functions	-	2.69%	-

**Table 3:** Summary of research on drilling fluid industry to which hybrid intelligence algorithms were applied.

Author (s)	Type of Study Conducted	Type of Hybrid	Performance Evaluation Criteria		
			RMSE	AAPE	R^2^
Owladeghaffari et al. (2009) [107]	Forecasting circulation losses of drilling fluids	utilizing self-organizing feature map fuzzy inference system (FIS)	-	-	-
Fatemeh et al. (2013) [108]	Kick assessment	Adaptive fuzzy neural networks	-	15.9753%	-
Zhou et al. (2016) [109]	Static hydrostatic pressure of drilling fluids under high-temperature and high-pressure conditions	PSO-BPNN	-	-	-
Ahmadi et al. (2018) [110]	Predicting the density of drilling fluids	PSO-ANN	0.0117	-	0.9964
		GA-FIS	0.3017	-	0.9397
Abdelgawad et al. (2019) [111]	Evaluating the rheological properties of drilling fluids	using adaptive differential evolution neural networks (SADE-ANN)	-	5%	0.95
Elkatatny et al. (2019) [112]	Predicting permeability to assess the reservoir protection capability of drilling fluids	SADE-ANN		5%	0.98
Rabault et al. (2019) [113]	Drilling fluid flow control	employing deep reinforcement learning-ANN	-	-	-
Alizadeh et al. (2021) [114]	Predicting the density of drilling fluids under high-temperature and high-pressure conditions	GA-LSSVM	0.0250	1.209%	0.996
		ICA-LSSVM	0.0174	0.625%	0.998
		PSO-LSSVM	0.0162	0.529%	0.999
Sabah et al. (2021) [115]	Forecasting circulation losses of drilling fluids	CSA-LSSVM	22.97 bbl/h	-	0.9424
		PSO-LSSVM	23.63 bbl/h	-	0.9391
		GA-MLP	25.26 bbl/h	-	0.9304
Davoodi et al. (2023) [116]	Evaluating the filtration wall-building properties of drilling fluids	utilizing CSA-multilayer extreme learning machines	PV: 0.6140 cP, YP: 0.7419 lb/100 ft^2^, FV: 0.6357 ml	PV: 2.0368%, YP: 3.6543%, FV: 8.0606%	PV: 0.9925, YP: 0.8672, FV: 0.9689
Huayang et al. (2023) [117]	Designing the upper limit of the safe drilling fluid density window	LSTM-BP	0.0152	-	0.9218
Liang et al. (2023) [118]	Investigating the rheological and wall-slip characteristics of drilling fluids	employing whale optimization–support vector regression	0.0013524 m/s	-	0.92906

**Table 4:** Comparative studies of various AI applications in the study of drilling fluid gel properties.

Author (s)	Study Conducted	AI Techniques Compared	Performance Evaluation Criteria		
			RMSE	AAPE	R^2^
Olutaya and Theodore (2011) [70]	Predicting the annular flow pattern of drilling fluid	ANN vs. SVM	-	-	ANN: 0.9028, SVM: 0.9528
Wang et al. (2012) [90]	Predicting the density of drilling fluid	ANN vs. SVM	ANN: 0.575, SVM: 0.117	ANN: 4.343%, SVM: 0.872%	ANN: 0.9889, SVM: 0.9994
Toreifi et al. (2014) [120]	Predicting drilling fluid loss and circulation	MNN vs. MLP	-	-	MLP: 0.745, MNN: 0.964
Jahanbakhshi and Keshavarzi (2015) [121]	Predicting drilling fluid loss and circulation	ANN vs. SVM	ANN: 76.2586, SVM: 27.6944	-	ANN: 0.7691, SVM: 0.9407
Hossein et al. (2018) [121]	Predicting the thermal conductivity of nanofluids	Self-organizing map (SOM) vs. Levenberg–Marquardt BP vs. LSSVM	-	-	SOM: 0.8812, LMBP: 0.8758, LSSVM: 0.9000
Ahmadi et al. (2018) [110]	Predicting the density of drilling fluid	FIS vs. PSO-ANN vs. GA-FIS	-	-	FIS: 0.7237, PSO-ANN: 0.9964, GA-FIS: 0.9379
Ahmadi (2023) [122]	Assessing wax deposition in the wellbore	GA-FIS vs. GA-ANN	GA-FIS: 0.0557, GA-ANN: 0.0480		GA-FIS: 0.9449, GA-ANN: 0.9681

**Table 5:** Strengths and weaknesses of Three numerical calculation methods.

Method	Finite Difference Method (FDM)	Finite Element Method (FEM)	Finite Volume Method (FVM)
Strength	Intuitive, mature theory. Selectable precision. Easy to program and parallelize. Fast computational speed.	Strong capability in handling complex boundaries. Selectable precision. High program versatility.	Clear physical significance. Suitable for irregular boundaries. Easy to parallelize.
Weaknesses	Tedious handling of complex boundaries. Presence of staircase errors.	High memory and computational requirements. Complex grid partitioning process. Not easily parallelizable.	Precision is not selectable.

**Table 6:** Summary of research on drilling fluid industry to which CFD was applied.

Author (s)	Type of Study Conducted	Algorithm Used/Simulator Based	Discretization Method	Findings
Dazhi and Tanner (1985) [131]	Investigating wall fluid characteristics	AXFINR program	FEM	when the flow index of drilling fluid is ≤0.5, the wall effect is not significant.
Butcher and Irvine (1990) [132]	Studying the drag force exerted on solid particles in drilling fluids	SIMPLER method	FDM	the drag coefficient obtained from numerical models is consistent with that from physical simulations
H. Scott Lane (1993) [133]	Simulating the invasion of water-based drilling fluids into formations and subsequent dissipation in the casing	Multiple Application Reservoir Simulator	FDM	capillary forces dominate the invasion process of drilling fluids in low-permeability formations.
Bush (1994) [134]	Influence of fluid characteristics on rock-carrying capacity.	POLYFLOW program, Viscoelastic model: Phan-Thien–Tanner fluid	FEM	The elongational characteristics of the fluid affect, to a large extent, the net response.
Blackery and Mitsoulis (1997) [135]	Creep of spheres in Bingham plastic fluids	Bingham constitutive equation modified with the Papanastasiou scheme	FEM	an increase in yield stress leads to an increase in drag coefficient.
Missirlis et al. (2001) [136]	Impact of wall fluid characteristics on the rock-carrying capacity of drilling fluids	Galerkin method	FEM and FVM	as the flow index increases, the drag coefficient initially increases and then decreases; when the flow index is 0, the drag coefficient tends towards 1.18, and this value is independent of the flow channel and the diameter of solid particles.
Jianghui et al. (2005) [137]	Simulating the process of drilling fluid invasion	UTCHEM	FDM	the rate of mudcake growth and the depth of filtrate invasion in high-permeability formations depend only on the properties of the mudcake itself, whereas in low-permeability formations, they are also influenced by formation factors.
Dhole et al. (2006) [138]	Characteristics of fluid flow	Guass-Seidel iteration	FVM	as the fluid flow index increases, the resistance also increases; however, when the flow index is >1, the increasing trend of resistance slows down
Salazar and Torres (2008) [139]	Simulating the invasion of water-based and oil-based drilling fluids into formations	Archie’s equation	FDM	the invasion depth of water-based drilling fluids is 15–40% longer than that of oil-based drilling fluids.
Bottero et al. (2010) [140]	Combining gas-liquid two-phase flow models with modern biofilm models to study the impact of proppant filling on gas velocity	Navier–Stokes equations and Cahn-Hilliard equation	FEM	hydrophobic proppants exhibit better dehydration effects than hydrophilic proppants
Prashant and Derksen (2011) [141]	Particle motion of solid particles in Bingham fluids	Lattice-Boltzmann technique	-	as the fluid yield stress increases, the settling velocity of particles decreases.
Gumulya et al. (2014) [142]	Simulating particle settling behavior in shear-thinning fluids	Volume of fluid method	FDM (implicit and central differencing scheme)	the flow field in shear-thinning fluids is divided into shear and unsheared fluid regions, with lower fluid viscosity in the shear region resulting in faster particle settling.
Gamwo and Kabir (2015) [143]	Investigating the influence of drilling fluid rheology and wellbore pressure on rock-carrying capacity	Eulerian–Eulerian multiphase model	-	the thickness of the borehole filter cake linearly increases with the pressure difference between the wellbore and the formation.
Mohammadzadeh et al. (2016) [144]	Effect of viscosity modifiers on the rock-carrying capacity of oil-based drilling fluids	Algebraic Slip Mixture model	FVM	drilling fluids with higher yield stress have stronger wellbore cleaning capabilities; higher concentrations of viscosifiers lead to increased rock-carrying capacity, but beyond the optimal concentration, the increase in capacity diminishes while pressure losses increase.
Akbari and Hashemabadi (2017) [145]	Simulation of annular drilling fluid flow	ASM model	FVM	when the drilling fluid flow rate is 0.75 m/s, the temperature increases from 70 °C to 90 °C at 0.1 MPa, resulting in a 2.08% reduction in cuttings transport volume; at 10 MPa pressure, the reduction is 1.63%.
Feng et al. (2018) [146]	Simulation of the migration and sealing process of lost circulation materials (LCM) in fractures	CFDEM	Discrete element method (DEM)	LCM with non-spherical shapes may induce multi-particle bridging, enhancing sealing efficiency.
Barbosa et al. (2019) [147]	Simulation of solid particle transport and deposition in drilling fluids	Eulerian–Lagrangian approach	DEM	using slow/medium flow rates and smaller diameter heavy particles is the most effective combination to mitigate fluid losses.
Xianyu et al. (2020) [148]	Prediction of the quantitative relationship between nanoscale particle parameters, fluid properties, and shale pore plugging efficiency	Ansys Fluent	DEM	a particle concentration of 1 wt% and fluid viscosity of 5 mPa·s are effective methods to enhance plugging efficiency.
Srawanti et al. (2021) [149]	Quantifying the impact of nanoscale zinc oxide on non-damaging drilling fluid rheology and circulation loss	Ansys Fluent	FVM	addition of nanoscale zinc oxide increases the shear stress and viscosity of non-damaging drilling fluids, enhancing viscoelastic solid properties at high temperatures.
Olalekan et al. (2022) [150]	Rheological study of drilling fluids containing thermochemical additives	COMSOL Multiphysics	FEM	the apparent viscosity of oil-based drilling fluids decreases with increasing temperature, while for water-based drilling fluids, the apparent viscosity increases within the range of 70–120 °C.
Zhu et al. (2023) [151]	Influence of mechanical shear on the anisotropy of fluid flow and LCM particle migration in fractures	CFD-DEM coupling method	FVM and DEM	appropriate shear in fractures with greater roughness aids in sealing drilling fluids.
Chong et al. (2024) [152]	Fracture sealing performance of LCM under high-temperature conditions	Volume-averaged Navier–Stokes equations	DEM	temperature increase typically leads to a decrease in LCM size, strength, and friction coefficient, as well as a decrease in drilling fluid viscosity.

## Data Availability

No new data were created.
